# Navigating Pediatric Capnography: A Comprehensive Review of Scope and Limitations

**DOI:** 10.7759/cureus.53289

**Published:** 2024-01-31

**Authors:** SreeHarsha Damam, Revat J Meshram, Amar Taksande, Sham Lohiya, Astha Khurana, Ankita Patel, Rahul Khandelwal, Ritwik Nath, Chaitanya Kumar Javvaji, Shikha Kakkat

**Affiliations:** 1 Pediatrics, Jawaharlal Nehru Medical College, Datta Meghe Institute of Higher Education and Research, Wardha, IND

**Keywords:** pediatric respiratory care, sidestream capnography, mainstream capnography, carbon dioxide measurement, respiratory monitoring, pediatric capnography

## Abstract

This review comprehensively explores pediatric capnography, a vital tool in contemporary respiratory monitoring. The overview encompasses the foundational principles of capnography, elucidating its real-time measurement of carbon dioxide (CO2) in respiratory gases. The review emphasizes its paramount role in pediatric care and underscores capnography's significance in detecting respiratory abnormalities and guiding timely interventions. The distinctions between mainstream and sidestream capnography, the key to understanding their applications, are meticulously outlined. Addressing the importance of ongoing research and education, the review advocates for a dynamic approach to refine guidelines and optimize capnography utilization in pediatric settings. The conclusion reflects on the scope and limitations of pediatric capnography, acknowledging its transformative impact while advocating for a judicious recognition of constraints. As we navigate the future of pediatric respiratory care, the synergy of research, education, and clinical application emerges as the cornerstone for advancing pediatric capnography to new horizons.

## Introduction and background

Capnography, a cornerstone in respiratory monitoring, provides a real-time assessment of exhaled carbon dioxide (CO2) levels. By graphically representing the concentration of CO2 over the respiratory cycle, capnography offers clinicians valuable insights into a patient's ventilatory and metabolic status [1]. In the pediatric context, where physiological parameters can change swiftly, capnography emerges as a critical tool for respiratory assessment. The unique dynamics of pediatric respiratory physiology, coupled with the challenges of early detection in this vulnerable population, underscore the significance of capnography [2]. From neonates to adolescents, applying capnography aids healthcare professionals in promptly identifying respiratory abnormalities, guiding therapeutic interventions, and ensuring optimal patient outcomes [3].

This comprehensive review aims to systematically examine the scope and limitations of capnography in the pediatric setting. This review seeks to equip healthcare practitioners with a nuanced understanding of when and how to leverage capnography in pediatric care by delving into the principles, equipment, waveform interpretation, and clinical applications. Additionally, we aim to shed light on the existing challenges and potential avenues for improvement, fostering a foundation for future advancements in pediatric capnography. Through this exploration, the review strives to contribute to the ongoing discourse on pediatric respiratory monitoring, emphasizing the pivotal role of capnography in enhancing the quality of care provided to the youngest members of our patient population.

## Review

Basics of capnography

Definition and Principles

Capnography involves quantifying the concentration of CO2 in respiratory gas, visually represented through the capnography waveform and quantitatively assessed via capnometry [4]. This non-invasive method is a standard for evaluating ventilation and has become integral to perioperative monitoring in pediatric anesthesiology [5]. Capnography proves valuable in diverse clinical scenarios, including confirming intubation, ensuring ventilation maintenance, evaluating the efficacy of cardiopulmonary resuscitation (CPR), and serving as an adjunct for monitoring sedated children and individuals with lower respiratory disease [5]. The capnography waveform illustrates the CO2 levels throughout each respiratory cycle phase, providing a comprehensive ventilation assessment. Simultaneously, the numerical value, end-tidal carbon dioxide (ETCO2), typically falls within the range of 35-45 mmHg [6]. This dual visual and numerical analysis approach enhances the precision of assessing respiratory function, making capnography an indispensable tool in pediatric care.

Components of Capnography Waveforms

Capnography waveforms encompass both a graphical representation and a numerical value. The numerical value, known as capnometry, signifies the partial pressure of carbon dioxide (PCO2) detected after exhalation, commonly called the ETCO2. Typically, ETCO2 falls within the range of 35-45 mmHg [6,7]. On the other hand, the capnography waveform provides a detailed depiction of the quantity of CO2 throughout distinct phases of the respiratory cycle and serves as a tool for evaluating ventilation [6,8,9].

This waveform is characterized by four phases: Phase I marks the inspiratory baseline, attributable to inspired gas containing low levels of CO2. Phase II signifies the commencement of expiration, arising as the anatomic dead space and alveolar gas mix. Phase III, the alveolar plateau, represents CO2-rich gas from the alveoli, typically exhibiting a positive slope indicative of a rising PCO2. Phase IV, a terminal upswing, may manifest towards the conclusion of Phase III [9]. Analyzing the waveform involves assessing five essential characteristics: height, frequency, rhythm, baseline, and shape [9]. This comprehensive analysis of capnography waveforms provides healthcare professionals with nuanced insights into the intricacies of respiratory dynamics and aids in evaluating ventilation parameters.

Normal Capnography Values in Pediatric Patients

In spontaneously breathing infants and children, the ETCO2 values typically fall within the 36-40 mmHg range, with an accepted normal range extending from 35 to 45 mmHg across all age groups [10,11]. Capnography has evolved into a standard for perioperative monitoring in pediatric anesthesiology. Its applications encompass confirming intubation, ensuring ventilation maintenance, evaluating the effectiveness of CPR, and serving as an adjunct for monitoring sedated children and those afflicted with lower respiratory disease [5,11,12]. Demonstrating efficacy as a non-invasive perioperative monitor, capnography proves invaluable in comprehensively assessing the physiological parameters and ensuring the safety of pediatric patients [5].

Indications for pediatric capnography

Respiratory Monitoring

Pediatric capnography, which involves measuring exhaled CO2, is a crucial tool in respiratory monitoring for children. Its applications are diverse, encompassing non-invasive assessment of the adequacy of mechanical ventilation, evaluation of ventilation integrity, and detection of occlusion and displacement of the endotracheal tube (ETT) [11]. Beyond these primary functions, capnography plays a pivotal role in confirming intubation, maintaining ventilation in both intubated and non-intubated children, monitoring the efficacy of CPR, and serving as an adjunct for monitoring sedated children and those with lower respiratory disease [11,12].

Moreover, capnography continuously monitors arterial CO2 tensions and is a reliable and instantaneous apnea monitor. This feature is particularly advantageous in the ventilatory management of neonates and children, where real-time information on respiratory status is critical [11,12]. As a non-invasive perioperative monitor, capnography has proven to be a valuable tool in comprehensively assessing the physiology and ensuring the safety of pediatric patients [5]. The multifaceted applications of capnography highlight its indispensable role in enhancing respiratory care for children across various clinical scenarios.

Ventilation Assessment

Capnography, the measurement of exhaled CO2, is valuable for evaluating ventilation in pediatric patients. This non-invasive method serves various critical functions, including monitoring the adequacy of mechanical ventilation, assessing ventilation integrity, and detecting occlusion or displacement of the ETT [11,12]. In addition to these primary applications, capnography proves instrumental in confirming intubation, ensuring ventilation maintenance in both intubated and non-intubated children, evaluating the effectiveness of CPR, and serving as an adjunct for monitoring sedated children and those with lower respiratory disease [11-13]. Moreover, capnography continuously monitors arterial CO2 tensions and functions as a dependable and instantaneous apnea monitor. This feature is particularly advantageous in the ventilatory management of neonates and children, providing real-time insights into respiratory status [11]. The multifaceted utility of capnography underscores its importance as an integral component in enhancing the comprehensive assessment and management of ventilation in pediatric patients across a spectrum of clinical scenarios.

Emergencies

Capnography is an invaluable diagnostic tool that informs and guides treatment decisions; however, it should be clarified that capnography, in and of itself, does not constitute a direct 'treatment' for the listed emergencies. Instead, it serves as a versatile tool for assessing and informing the treatment of ventilation, perfusion, and metabolic emergencies in pediatric patients [10]. The continuous measurement of exhaled CO2 provides real-time insights into the patient's respiratory status, enabling healthcare providers to promptly identify and address abnormalities in ventilation and metabolic processes during emergencies. In respiratory distress or metabolic crises, capnography is crucial in tailoring interventions to the child's specific needs, ensuring a targeted and effective approach to emergent situations.

Identification of sick children: Utilizing capnography waveform facilitates the early identification of sick children, distinguishing them from healthy counterparts. This capability is particularly crucial for emergency medical services (EMS) providers, enabling timely and accurate interventions before the child's condition deteriorates [10]. Early recognition of physiological disturbances through capnography enhances the ability to initiate appropriate medical interventions promptly, potentially preventing further decompensation and optimizing patient outcomes.

Monitoring the effectiveness of CPR: Capnography is pivotal in improving the quality of chest compressions during CPR. Capnography guides healthcare providers by providing real-time feedback on ventilation and perfusion, ensuring that chest compressions are effective and aligned with resuscitation guidelines [14]. This dynamic feedback loop enhances the precision of CPR delivery, contributing to increased chances of successful resuscitation in pediatric patients.

Optimizing procedural sedation is crucial, particularly in the emergency department, where capnography is pivotal. Specifically, capnography is valuable for optimizing the administration of sedative agents in the pediatric population [12]. In addition to monitoring the levels of ETCO2 continuously, it explicitly aids in detecting apnea. This capability assists healthcare providers in maintaining a delicate balance between achieving appropriate sedation levels and ensuring the patient's respiratory safety during procedural sedation procedures. Including explicit apnea detection further enhances the safety and efficacy of procedural sedation in pediatric emergency care settings.

Monitoring airway management: Capnography is a non-invasive and indispensable tool for airway management in emergencies involving pediatric patients [11]. It provides continuous feedback on the effectiveness of ventilation, aiding healthcare providers in ensuring proper airway patency and function. This is particularly critical during interventions such as intubation or ventilation support, where immediate feedback on the patient's respiratory status is essential for optimal care. Procedural sedation, also referred to as procedural sedation and analgesia (PSA), entails the administration of sedatives or dissociative agents, with or without analgesics, to induce a state that enables patients to tolerate uncomfortable procedures while maintaining cardiorespiratory function [15,16]. This approach serves various objectives, encompassing patient safety, mitigating pain and anxiety, minimizing patient movement during a procedure, and enhancing the likelihood of procedural success [15-18]. 

Equipment and technology

Types of Capnography Devices

Colorimetric capnography: Colorimetric capnography utilizes a colorimeter to quantify the concentration of CO2 in exhaled air by analyzing the absorbance of light at different wavelengths [12]. This method visually represents CO2 levels based on color changes, offering a straightforward yet effective means of assessing ventilation.

Electrochemical capnography: Electrochemical capnography employs an electrochemical sensor to measure the PCO2 in exhaled air. This method is widely utilized in clinical settings because of its precision and user-friendly features [12]. The electrochemical sensor provides accurate real-time data, contributing to its popularity in medical applications.

Capnography waveform: Capnography waveform goes beyond measuring CO2 levels by capturing the entire waveform of exhaled CO2. This technology offers additional insights into the respiratory system, aiding in the early detection of potential issues [19]. Capnography waveform holds promise for significant utility in prehospital settings, where detailed data can be precious [19].

Capnometry: Capnometry, akin to capnography, employs a specialized device to quantify CO2 levels in exhaled air [19]. Often used in conjunction with pulse oximetry, capnometry monitors oxygenation and ventilation in pediatric patients, providing a comprehensive respiratory assessment [20].

Combination capnometer and pulse oximeter devices: Devices combining capnometry and pulse oximetry measure both CO2 levels and oxygen (O2) saturation in the blood. This integrated approach offers a holistic view of a patient's respiratory and circulatory status [21]. Such combination devices are versatile tools utilized across various medical settings, including pediatric anesthesiology, emergency departments, and intensive care units. Capnography devices employing these diverse methods find extensive application in clinical practice. They play crucial roles in monitoring the efficacy of ventilation, confirming the accurate placement of endotracheal tubes, and guiding both non-invasive and invasive ventilation strategies [12,20]. These technologies significantly enhance patient care and safety in diverse healthcare environments.

Differences Between Mainstream and Sidestream Capnography

Mainstream and sidestream capnography are two methods for measuring CO2 levels in respiratory gases. Each has distinct advantages and limitations, as described in Table 1 [22].

**Table 1:** Mainstream and sidestream capnography feature CO2: carbon dioxide

Feature	Mainstream capnography	Sidestream capnography
Sensor location	Directly in the main airway (e.g., at the end of an endotracheal tube or an adapter)	Away from the main airway, samples of gas from the breathing circuit
Sampling method	Measures CO2 in real-time as it occurs	Samples of gas from the breathing circuit with a time lag
Size and weight	Smaller and lighter sensor	The sampling tube and aspirator mechanism add weight and complexity
Dead space	Adds minimal dead space	Introduces additional dead space in the breathing circuit
Infection control	Potentially more infection-resistant	The sampling line may require regular replacement, with the potential for contamination
Temperature and humidity effects	Less affected by changes in temperature and humidity	Changes in temperature and humidity along the sampling line can affect accuracy
Applicability	Often used in intubated patients and during surgery	More versatile, applicable in various settings, including non-intubated patients

Challenges and Considerations in Pediatric Capnography Equipment

Using capnography in pediatric patients presents specific challenges and considerations concerning equipment and technology. Although capnography has become a standard tool for monitoring the physiology and safety of children, there are methodological and technical constraints associated with neonates and small infants when using presently available equipment [23]. These limitations encompass factors such as the impact of small airways on gas exchange, the heightened influence of dead space on measurements, and the necessity for specialized equipment to ensure accurate and reliable monitoring in this population [23].

Notwithstanding these challenges, capnography holds diverse clinical applications in pediatric patients, serving purposes such as confirming intubation, maintaining ventilation, assessing the effectiveness of CPR, and functioning as an adjunct for monitoring sedated children and those with lower respiratory disease [12]. The continuous monitoring of capnography can swiftly furnish objective information about changes in a patient's ventilatory status, contributing to enhanced patient care and safety [12].

While both the technology and understanding of capnography have advanced, and a growing body of evidence supports its use in the literature, it remains imperative to acknowledge the existing limitations and tailor considerations to the specific needs of pediatric patients. This ensures the accurate and reliable application of capnography within this unique population [12,23].

Pediatric capnography waveforms

Interpretation of Capnography Waveforms

Normal capnogram: A typical capnogram follows a standardized pattern encompassing four distinct phases: Phase I, known as the inspiratory baseline; Phase II, identified by the expiratory upstroke; Phase III, characterized by the alveolar plateau; and Phase IV, denoting the end-tidal phase. The normal waveform is recognized by its rectangular shape, and the ETCO2 value aligns within the expected range [9]. This baseline representation provides a fundamental understanding of a healthy respiratory cycle.

Abnormal waveforms: Anomalies in capnography waveforms can signal specific issues. Instances such as a floating baseline, incomplete exhalation, or irregular shape may point to problems like rebreathing, air trapping, or equipment malfunction [24,25]. Recognition of these deviations is critical for the timely identification and resolution of underlying concerns during patient monitoring.

Analyzing waveform characteristics: Interpretation of capnography waveforms necessitates a thorough examination of critical characteristics, including height, frequency, rhythm, baseline, and shape. These alterations can offer valuable diagnostic insights, prompting healthcare professionals to consider potential respiratory challenges or interventions [9]. A meticulous analysis of these parameters enhances the precision of waveform interpretation.

Clinical applications: Capnography waveform interpretation finds diverse clinical applications, aiding in diagnosing conditions such as esophageal intubation, bronchus intubation, and cardiogenic oscillations. Additionally, it proves instrumental in troubleshooting ventilator-related issues and monitoring patient responses to interventions [9]. A nuanced understanding of these aspects of capnography waveform interpretation is indispensable for healthcare professionals involved in the care of patients, especially those undergoing respiratory monitoring and management. This knowledge equips providers to make informed decisions and optimize patient care in various clinical scenarios.

Normal and Abnormal Patterns

Capnography waveforms are a valuable source of information for assessing a patient's ventilation, perfusion, and metabolism. A typical capnography waveform has an almost rectangular appearance and is characterized by four main phases, providing insights into the movement of CO2 within the airways [4,26]. These phases include the inspiratory baseline, where minimal CO2 is detected, resulting in a flat waveform (Phase I); the rapid rise as alveolar gas replaces anatomical dead space (Phase II); the alveolar plateau, representing the highest concentration of exhaled CO2 (Phase III); and the end-tidal phase, marking the transition from exhalation to inspiration (Phase IV).

Abnormal capnography waveforms can indicate various conditions, such as airway obstruction, rebreathing, hypoventilation, hyperventilation, or esophageal intubation [4,7,27]. The detection of abnormalities involves analyzing the height, shape, frequency, rhythm, speed of change of the waveform, and numeric values [4]. Therefore, understanding how to read and interpret capnography waveforms is crucial for healthcare professionals to ensure patient safety and well-being [28].

In clinical settings, proficiency in interpreting capnography waveforms is essential, enabling healthcare professionals to make timely interventions and optimize patient care. The nuanced insights provided by capnography contribute significantly to monitoring respiratory dynamics and promptly addressing potential issues, ultimately enhancing patients' overall safety and well-being.

Case Studies Illustrating Waveform Interpretation in Pediatric Patients

Altered mental status: Capnography proves to be a valuable tool in recognizing altered mental status among pediatric patients, especially in scenarios involving seizures, head injuries, and overdoses. The primary focus is on its contribution to ensuring optimal ventilation in the context of altered medical conditions. By meticulously assessing capnography waveforms and monitoring ETCO2 levels, healthcare professionals gain crucial data that aids in promptly recognizing and managing conditions impacting neurological function [10]. The emphasis here is on the role of capnography in providing real-time insights into respiratory dynamics, contributing to more effective interventions and overall patient care in cases of altered mental status.

Confirmation of ETT placement: Capnography is recognized as a valuable tool in confirming the accurate placement of an ETT. While the literature supports its usefulness, the term 'critical' may require nuanced consideration based on the population under discussion. It should be noted that the application of capnography in confirming ETT placement is more commonly emphasized in adult and pediatric populations than in infants or neonates. By detecting the presence of CO2 in exhaled air, capnography contributes significantly to preventing misplacement and mitigating the risk of associated complications. This application remains particularly crucial in ensuring the safety of pediatric patients undergoing intubation procedures [12].

Monitoring ventilation: Capnography is indispensable for monitoring ventilation adequacy in intubated and non-intubated pediatric patients. Its utility extends to various clinical scenarios, including anesthesia administration and emergencies. Real-time feedback from capnography aids healthcare providers in optimizing ventilation strategies for improved patient outcomes [10,29].

Assessment of circulation: Capnography provides indirect information about circulation, adding to its significance in monitoring the effectiveness of CPR and assessing circulatory status. The real-time data offered by capnography aids in gauging the success of resuscitative efforts, contributing to more informed decision-making during critical events [30].

Non-invasive monitoring: Capnography has demonstrated its utility in perioperative and non-perioperative settings as a non-invasive monitor of a child's physiology and safety. This non-intrusive approach makes capnography particularly valuable in ensuring continuous monitoring without compromising the comfort or well-being of pediatric patients [10,29]. Collectively, these applications underscore the paramount importance of capnography in pediatric care. Capnography emerges as a versatile and crucial tool for confirming ETT placement, assessing neurological conditions, optimizing ventilation, or providing indirect insights into circulation. Its non-invasive nature further enhances its applicability in diverse clinical scenarios, ensuring the safety and well-being of children across a spectrum of healthcare settings.

Limitations and challenges

Factors Affecting Accuracy in Pediatric Capnography

Capnography technique: The accuracy of capnography measurements can be compromised by technical factors. Improper placement of the sampling site, whether distal or proximal, and the type of capnometer used can introduce inaccuracies in the recorded data [31]. Attention to these technical aspects is crucial to ensure reliable and precise capnography readings, especially in dynamic clinical settings.

Patient factors: Ventilatory parameters, particularly in very low birth weight newborns, present unique challenges for capnography. The considerably lower volume of exhaled CO2 and reduced tidal volumes in this population can affect the sensitivity of capnography, requiring careful consideration and potential adjustments for accurate monitoring [32].

Clinical factors: Several clinical factors can contribute to discrepancies between arterial and transcutaneous values of CO2, impacting the overall accuracy of capnography readings [32]. The dynamic nature of clinical conditions may introduce variability that healthcare providers must account for when interpreting capnography data for comprehensive patient assessment.

Environmental factors: Environmental conditions, such as temperature, can influence the precision of capnography measurements. Awareness of these environmental factors is essential for maintaining the accuracy of the equipment and ensuring reliable data collection [5]. Attention to the impact of external variables contributes to the overall quality and dependability of capnography in clinical practice.

Limitations in pediatric patients: Skepticism surrounding capnography often stems from challenges in obtaining accurate measurements, particularly concerning ventilatory parameters [11]. Despite these challenges, capnography has proven to be a valuable non-invasive perioperative monitor for assessing the physiology and safety of children [5]. However, acknowledging the limitations inherent in pediatric populations is essential to understanding the data and comprehensively enhancing overall patient safety.

Age-Specific Considerations

Neonates and infants: Utilizing capnography in intubated neonates and infants presents challenges, primarily stemming from lower exhaled CO2 and tidal volumes. In this population, the difficulty in obtaining venous access is compounded by their small size and limited visible peripheral veins, further complicating the comprehensive monitoring of ventilatory parameters [33]. The intricacies of capnography use in these cases necessitate a nuanced approach to ensure accurate assessment and patient safety.

Toddlers: Their distinct physiological characteristics influence capnography monitoring in toddlers. Toddlers typically exhibit a higher respiratory rate than older children, posing challenges for capnography monitoring. Their blood pressure values also tend to be lower, introducing considerations for accurate and meaningful data interpretation [33]. Addressing these age-specific factors is imperative for practical capnography application in the toddler population.

School-age children and adolescents: As children progress through different developmental stages, changes in vital signs, including respiratory rate, pulse, and blood pressure, necessitate adjustments in capnography monitoring. A tailored approach is crucial to accurately assess respiratory and cardiovascular status in school-age children and adolescents [33]. Adapting monitoring strategies to the evolving physiology of older children is essential for reliable data interpretation.

Feasibility during specific procedures: The feasibility of capnography during certain procedures, such as bronchoscopy or the repair of facial lacerations, may be compromised if the patient is agitated or uncooperative during the initial phases of sedation. In such cases, practical considerations may dictate the placement of the capnography monitor once the child becomes sedated, ensuring optimal monitoring conditions and accurate data collection [34].

Monitoring during sedation: Capnography is deemed essential for almost all deeply sedated children due to the heightened risk of airway/ventilation compromise. However, its feasibility may be limited if the patient exhibits agitation or uncooperativeness during the initial phases of sedation. In such instances, a strategic approach involves placing the capnography monitor once the child reaches a sedated state, striking a balance between monitoring necessity and patient comfort [34].

Common Challenges in Neonates, Infants, and Older Children

Pediatric capnography encounters notable challenges across varying age groups, encompassing neonates, infants, and older children. The conventional technology employed in capnography faces limitations that impact its reliability, including sensitivity to changes in temperature and the necessity for frequent zeroing and recalibration processes [23,35]. In neonates, the unique physiological characteristics, such as the low volume of exhaled CO2 and diminished tidal volumes, present significant hurdles for achieving accurate measurements [23]. Moreover, the complexities of airway management and the elevated rates of intubation failure in neonatology compound the challenges associated with capnography implementation in this population [36]. While capnography is a routine tool for patient transport in older children and adults, its application in neonatal transport remains a subject of ongoing discussion and consideration [37]. Neonates' distinct physiological and clinical intricacies contribute to the deliberation surrounding the optimal utilization of capnography during their transport. This underscores the need for continuous research endeavors and technological advancements to refine and enhance the application of capnography in pediatric and neonatal care settings. These challenges emphasize the importance of sustained efforts in research and innovation to address the specific needs of pediatric patients across various age groups. Ongoing advancements in capnography technology hold the potential to overcome existing limitations and improve its efficacy in monitoring and enhancing the care of neonates, infants, and older children, ultimately contributing to advancements in pediatric and neonatal healthcare practices.

Clinical applications

Use in Various Clinical Settings (Emergency Department, Intensive Care Unit, General Pediatrics)

Pediatric capnography demonstrates diverse clinical applications across different medical settings, including the emergency department, intensive care unit, and general pediatrics. It is valuable for assessing and monitoring pediatric patients in these environments. Capnography plays a crucial role in confirming intubation, maintaining ventilation in intubated and non-intubated children, monitoring the effectiveness of CPR, and serving as an adjunct for monitoring sedated children and those with lower respiratory disease [12]. Beyond these general applications, it has specific diagnostic roles for pediatric patients dealing with congenital heart disease, reactive airway disease, neurologic emergencies, and metabolic derangement [2].

As a non-invasive method for measuring exhaled CO2, capnography provides essential insights into critically ill pediatric patients' breathing and circulation status [2]. Despite its proven effectiveness, capnography's full diagnostic and therapeutic potential must be consistently recognized, leading to its underutilization in pediatric practice [11]. Therefore, enhancing awareness regarding the diverse applications of capnography in pediatric care is paramount for improving patient outcomes. Acknowledging and incorporating the multifaceted benefits of capnography can significantly contribute to the comprehensive care and management of pediatric patients in various healthcare settings.

Integration With Other Monitoring Tools

Capnography is a versatile tool seamlessly integrated into various medical equipment, including defibrillators, anesthesiology machines, and patient monitoring systems [1]. Its real-time monitoring capability allows healthcare providers to continuously assess a patient's ventilation status, enabling timely identification of potential breathing complications and facilitating swift adjustments in clinical management [1]. Capnography devices come in handheld portable forms or are incorporated as modules or components within other medical equipment [1].

This monitoring technology proves invaluable for patients across all age groups, from neonates to adults, offering critical insights into ventilation success, respiratory effort levels, and the status of ventilated patients [38]. In addition to confirming intubation success, capnography is crucial for monitoring non-intubated patients and providing assessments of ventilation and perfusion [38]. The increasing recommendation for capnography in confirming ETT insertion and continuously monitoring exhaled CO2 in intubated patients underlines its expanding role as a standard monitoring tool, particularly in enhancing safety for intubated patients [39]. The continuous monitoring of quantitative capnography waveform is becoming a standard practice for all intubated patients [39]. Capnography's integration with other monitoring tools offers a comprehensive assessment of pediatric patients' ventilation, perfusion, and metabolism. This collaborative approach improves patient care and safety and positions capnography as an essential component in the continuum of monitoring technologies for diverse healthcare applications [1,38,39].

Pediatric-Specific Guidelines and Recommendations

Off-label drug use in pediatric guidelines: In a detailed analysis encompassing 66 pediatric guidelines with a total of 605 recommendations, it was revealed that 23.1% of these recommendations were assessed using the Grading of Recommendations, Assessment, Development, and Evaluations (GRADE) system. Within this subset, 26.4% were graded as strong, albeit supported by evidence classified at levels C or D. This underscores a notable prevalence of off-label drug use within the pediatric context, highlighting the imperative for careful consideration and evidence-based practices in pediatric healthcare [40].

Clinical practice guideline for the evaluation and treatment of children and adolescents with obesity: The American Academy of Pediatrics (AAP) introduced the initial edition of this guideline, emphasizing a child-centric approach that transcends specific healthcare settings. Aimed at informing pediatricians and other healthcare providers, these guidelines focus on practical strategies for preventing and treating obesity in children and adolescents. The comprehensive nature of these guidelines addresses a critical health concern among the pediatric population [41].

Recommendations for preventive pediatric healthcare: Collaborative efforts between the AAP and Bright Futures have resulted in guidelines offering preventive pediatric healthcare recommendations. Emphasizing continuity of care and personalized guidance for each child and family, these guidelines underscore the holistic approach required for promoting and maintaining the health of pediatric patients [42].

Pediatric Intensive Care Unit (PICU) Environment and Early Mobility (PANDEM) guidelines for infants and children: Published by the Society of Critical Care Medicine (SCCM), the PANDEM guidelines adopt a broad-spectrum approach to the care of critically ill infants and children. Focusing on seven crucial domains of care, including pain, sedation/agitation, iatrogenic withdrawal, neuromuscular blockade, delirium, PICU environment, and early mobility, these guidelines contribute to a comprehensive framework for managing critical pediatric cases [43].

Pediatric practice guidelines: This textbook is a valuable resource, offering evidence-based pediatric guidelines tailored for seasoned and novice clinicians. Concentrating on prevalent pediatric conditions encountered in primary care settings, these guidelines emphasize the pivotal role of nurses in pediatric health promotion. Presented in an easily accessible outline/bulleted format, the guidelines facilitate quick access to crucial information, promoting efficient and informed decision-making in pediatric healthcare [43].

Training and education

Importance of Healthcare Provider Training in Pediatric Capnography

Increased use in perioperative monitoring: Capnography has emerged as a standard tool for perioperative monitoring in pediatric anesthesiology, gradually extending its applications beyond the perioperative setting. Its utility expands to various scenarios within pediatric care, showcasing its versatility and significance in enhancing patient safety and outcomes [5].

Variability in dissemination and acceptability: While capnography in pediatrics is rising, its dissemination and acceptance across all facets of pediatric care exhibit variability. Adopting capnography practices may differ among healthcare settings, highlighting the need for more consistent and widespread integration of this valuable monitoring tool throughout pediatric healthcare [5].

Improved patient safety and outcomes: Capnography has demonstrated its effectiveness as a non-invasive perioperative monitor, contributing significantly to pediatric patients' safety and physiological assessment. By identifying individuals at risk for complications, capnography is a valuable tool guiding clinicians in optimizing patient care and improving overall outcomes [5].

Education and support: The literature underscores the pivotal role of education in successfully implementing and utilizing capnography. Healthcare providers require comprehensive training in capnography to ensure accurate data interpretation and optimal patient care. A well-informed and trained healthcare workforce is essential for maximizing the benefits of capnography in pediatric settings [44-47].

Quality improvement and patient safety: Capnography is crucial in enhancing the quality of chest compressions during CPR, minimizing interruptions, and potentially improving outcomes. Adequate training in pediatric capnography is essential for healthcare professionals to respond effectively during emergencies, contributing to better patient outcomes [14].

Familiarity with capnography equipment: Healthcare providers must be well-versed in the equipment and techniques employed in capnography to ensure precise monitoring and timely intervention. Familiarity with capnography equipment enhances the efficiency and accuracy of monitoring, reinforcing its role as a valuable tool in various healthcare scenarios [14].

Incorporation Into the Medical Education Curriculum

Integrating pediatric capnography into medical education curricula is paramount for advancing patient care, particularly within emergency medicine. Platforms like CapnoAcademy provide accessible online training dedicated to educating EMS clinicians on the effective utilization of capnography monitoring in prehospital environments [48]. Additionally, educational content such as the Pediatric Video Tutorial by Pedi-Ed-Trics Emergency Medical Solutions offers valuable insights into pediatric capnography through video tutorials [47].

Moreover, a study highlighted by the National Center for Biotechnology Information (NCBI) underscores the pivotal role of capnography in the pediatric emergency department. This research emphasizes the ongoing need for education and advocacy to enhance the integration of capnography, ultimately improving patient outcomes [14]. These educational resources and research findings underscore the importance of incorporating pediatric capnography training into medical education curricula. This proactive approach aims to elevate the quality of patient care, particularly in the emergency treatment of pediatric patients.

Safety considerations

Patient Safety During Capnography Monitoring

Implementing capnography monitoring has significantly enhanced patient safety when administering PSA to pediatric patients [49,50]. Considering the heightened risk of airway/ventilation compromise in deeply sedated children, capnography is recommended for nearly all cases [34]. This monitoring tool proves particularly valuable when observation is challenging, such as during MRI procedures or in dimly lit environments [34]. Capnography's capability to detect hypoventilation and apnea before these issues become clinically apparent or register on pulse oximetry makes it a crucial but underutilized asset in pediatric care [51]. Additionally, it offers immediate identification of ventilatory circuit disconnections in pediatric patients, allowing for instantaneous intervention before changes in O2 and CO2 levels occur [50]. In the emergency department, capnography is a highly effective continuous ventilation monitor, providing immediate indications of apnea or severe hypoventilation [50]. Despite its evident utility, capnography remains underutilized, emphasizing the need for broader recognition and integration of this technology to enhance patient safety in pediatric settings.

Addressing Potential Complications and Troubleshooting

Decreased utilization of blood gases: Implementing continuous capnography has demonstrated a notable reduction in the reliance on blood gases. This shift towards continuous, non-invasive monitoring of CO2 levels is a valuable alternative, providing ongoing assessment of ventilation adequacy and contributing to enhanced patient safety [52].

The shape of the capnography waveform: The shape of the capnography waveform serves as a diagnostic indicator for respiratory issues. This aspect of capnography becomes a valuable tool in assessing and managing respiratory distress by offering real-time feedback on the patient's response to treatment. It allows healthcare providers to tailor interventions based on immediate and dynamic information [53].

Patient safety benefits: Studies have underscored the positive impact of capnography monitoring on patient safety during procedural sedation. By aiding in the early detection of adverse events, capnography provides consistent evidence of safety enhancements in clinical settings. This emphasizes its role as a proactive measure in ensuring patient well-being [50].

Feasibility and limitations: While capnography proves highly beneficial, its feasibility may be compromised in specific circumstances, such as when patients are agitated or uncooperative during the initial phases of sedation or specific procedures. Acknowledging these limitations is crucial, and documenting situations where capnography may not be suitable helps guide its appropriate use [34].

Troubleshooting: Regularly checking the capnogram for abnormalities, including increases, decreases, or irregular waveforms, is essential for effective troubleshooting. The ability to interpret these findings provides healthcare professionals with valuable insights into potential issues, enabling prompt and targeted interventions [53].

## Conclusions

This comprehensive review has systematically examined the landscape of pediatric capnography, encapsulating its fundamental principles, applications, and nuanced considerations. The key points emphasized the real-time nature of capnography, its pivotal role in pediatric respiratory monitoring, and the intricacies associated with waveform interpretation and equipment selection. Moving forward, the importance of ongoing research and education in pediatric capnography cannot be overstated. The dynamic nature of pediatric physiology underscores the need for continuous exploration and refinement of guidelines to optimize capnographic monitoring. Healthcare professionals are encouraged to engage in ongoing education to stay abreast of technological advancements and evolving insights into pediatric respiratory patterns. Lastly, reflecting on the scope and limitations of pediatric capnography, this review underscores the critical balance between recognizing its transformative potential and acknowledging its inherent constraints. By maintaining a cautious and informed approach, healthcare providers can harness the strengths of capnography while navigating its limitations judiciously. As we chart the future of pediatric respiratory care, the symbiosis of research, education, and clinical application will propel pediatric capnography to new heights, ensuring its continued efficacy in delivering precise and tailored respiratory management for our youngest patients.
